# Velocity dependent up-winding scheme for node control volume finite element method for fluid flow in porous media

**DOI:** 10.1038/s41598-020-61324-4

**Published:** 2020-03-10

**Authors:** Abdul Salam Abd, Ahmad Abushaikha

**Affiliations:** Division of Sustainable Development, College of Science and Engineering, Hamad Bin Khalifa University, Education City, Qatar Foundation, P.O. Box 34110, Doha, Qatar

**Keywords:** Engineering, Applied physics, Fluid dynamics

## Abstract

We present a novel velocity based up-winding scheme for the node control volume finite element (NCVFE) method. The NCVFE method solves for the pressure at the vertices of elements and a control volume mesh is constructed around them; where the advection of fluids is modelled. Therefore, each element shares several control volumes, and traditionally the fluid saturations used in calculating the mobilities over each element  −  hence updating pressure  −  are arithmetically weighted. In this paper, we use the velocity vector to allocate the upstream direction of the fluid flow in each element and use the upstream fluid saturation in calculating the mobility needed for the pressure equation. We test his novel approach using triangle and tetrahedron elements, and we show that it produces more accurate fluid saturation profiles than the traditional approach. The method can easily be implemented in current NCVFE simulators.

## Introduction

Upstream mobility calculation in modelling multi-phase flow in porous media is widely accepted and considered stable and accurate. This approach uses fluid phase information from the upstream cell/grid for the calculation of fluxes when modelling the transport of fluids to solve fluid dynamic problems. Aziz and Settari^1^ applied the method for finite difference based reservoir simulators to model multi-phase fluid flow in petroleum reservoirs. The method is similar to the donor cell up-wending scheme applied in the finite volume method^2^. Sammon^3^ and Brenier and Jaffre^4^ showed that the method is consistent, convergent and well-defined (for one dimensional cases). Higher order schemes of the method were also applied^5,6^.

The equations governing multi-phase fluid flow in subsurface porous media are of mixed characteristics. The pressure equation is elliptic and the transport equations of fluids are hyperbolic. These governing equations have been implemented in various studies to predict fluid flow in naturally fractured reservoirs^7^ and even in pore-scale modelling of heterogeneous porous media^8^. Moreover, the hydrology community relies on similar numerical models to simulate groundwater movement, contaminants transport in aquifers chemical reactions between minerals^9,10^. The numerical method used to solve these equations is the core of reservoir simulation. Such methods discretize the equations from partial differential form to algebraic equations in space and time. Two popular categories for numerical discretization of flow problems amongst the reservoir community are the finite volume method (FVM) and finite element method (FEM), where the latter is divided into three approaches: sadfss. FVM is a simple and common numerical method in the computational flow dynamics community. It is used discretize the reservoir fluid flow equations and is well adapted for conservation laws. However, its geometrical flexibility is limited since it is dependent on the level of orthogonality between the cells^11^. This is considered a large handicap as it makes it very difficult to model the complex geometry of hydrocarbon reservoirs and requires very fine meshes to obtain reasonable results which is computationally very expensive^11,12^.

In FEM, the domian is divided into a set of elements that communicate through interpolation function. The governing equations are written in integral formulation and forms a system of local algebraic equations written in matrix form. This matrix is solved to obtain the pressure value at the nodes which can be located at the vertices, centroid, or interfaces of the elements. FEM represent the domain using various shapes of elements: line, triangle, tetrahedron, prism, hexahedron and others. This large library of elements gives the method the ability and the strength to model domains with large complexity including discrete fracture models^13^. To solve for complex reservoir engineering problems, many advances have been made over the past 50 years, which can be categorized into three main approaches. In the Coupled upwind-weighted finite element method (CUFE) approach, we solve for the fully coupled flow equations, saturation and pressure equations with up-streamed mobility values^14–16^. The interfaces between neighboring elements do not necessarily have to be to be orthogonal^12^ and and the accuracy of the method is mesh dependent^17–20^. Moreover, Newton’s method is needed to solve for the coupled equations which increase the computational cost deeming it as a drawback^12,21^.

In the Node control volume finite element method (NCVFE) approach, the saturation and pressure equations are decoupled while imposing a secondary mesh around the finite element nodes (vertices). This method was first introduced to the computational flow dynamics community by^22^. In NCVFE, we we use the Galerkin finite element method to solve for the pressure on the element’s vertices, while the saturation is solved explicitly on the control volumes. After that, the the phase velocities are computed using Darcy’s law and the pressures. Finally, the continuity equation is used to calculate the advection and transport of fluid on the node control volumes^12,19,21,23–25^.

The NCVFE can be used to model complex structures while yielding better simulation results than CUFE methods. However, the capillary pressure can only vary in a continuous fashion for the method to be accurate^12,21^. Moreover, adaptive mesh in space and time can be used easily in NCVFE, as proposed by^11^, and the use of discrete fracture models (DFM) in 2D and 3D meshes^12,26,27^. However, a main drawback of NCVFE is the fact that the control volume mesh is constructed around the nodes and the material properties are assigned on elements. This entails that there is a loss of physical accuracy and artificial fluid smearing when modelling multi-phase flow in highly heterogeneous and fractured reservoirs.

In order to overcome this drawback of artificial smearing, continuous fluxes across element interfaces need to be assured. This concept is applied in the mixed finite element (MFE) method^25,28–32^. In this methods, two or more primary variables are solved where in fluid flow problems, the MFE method solves the pressure and the velocity fields simultaneously. The continuity of fluxes across the elements’ interfaces is guaranteed by vectorial interpolation functions, i.e. Raviart-Thomas^33^. The fluxes are solved on the element interfaces and the pressure is solved on the centroid of element in a fully implicit scheme similar to^34^. The main difference between MFE and NCVFE methodsis that MFE approximates flow variables more accurately and realistically than NCVFE in small highly heterogeneous permeability cases^35^.

**Figure 1:**
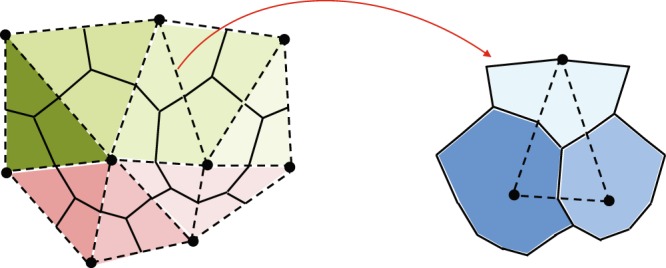
Node control volume mesh for triangular elements. The material properties are defined on the elements and the pressure and transport variables are computed on the control volumes.

In our work, we utilize node control volume finite element (NCVFE) method. As mentioned earlier, the elliptic and hyperbolic equations are decoupled, and they are solved on two different meshes. The former is solved using the Galerkin method on a finite element mesh and the latter using the finite volume method on the node control volume mesh^12,36^, see Fig. 1. These two meshes are not aligned since the control volume mesh is constructed around the vertices of the finite element mesh. Therefore, each element shares several control volumes; hence several values of the transport information (i.e. fluids saturation, see Fig. 1), and traditionally these fluid saturations are arithmetically weighted over each element to be used for the pressure equation, as we show in Section 3. While the transport equations use the upstream method to compute the phase fluxes between the node control volumes. This mismatch in the mobility calculation between the pressure and transport equations along with the misalignment of the corresponding meshes produce inaccurate fluid saturation profiles. Especially, in heterogeneous media since the material properties are imposed on the elements and the transport values are computed on the control volumes, as discussed by Abushaikha *et al*.^37^. Higher order schemes for the mobility calculation between the control volumes have also been applied^38,39^.

The objective of this paper is to present and investigate a new mathematical formulation for mobility upstreaming over mesh elements in a reservoir simulation. The method entails a velocity vector that starts from the element’s interface and is piece-wise constant in first order elements. The vector is used to approximate the upstream saturation in the given element. This results showed accurate saturation profiles when compared to the traditional arithmetic weighting method,even in multiphase flow scenarios. Furthermore, the UMC method can be easily implemented in current NCVFE based simulators. The paper is organized as follows. In Sections 2 and 3, the governing equations and the Node Control Volume Finite Element method are introduced. In Section 4, the upstream mobility calculation for NCVFE is described, and in Section 5, numerical tests are presented. Finally, conclusions are given in Section 5.

## Governing equations

We consider two-phase immiscible fluid flow of water and oil in heterogeneous porous media described by the continuity equation and Darcy’s law^40^. We assume a slightly compressible rock. The mass balance for the fluid phase *α* is,

1 $$\frac{\partial (\phi {\rho }_{\alpha }{S}_{\alpha })}{\partial t}=-\nabla \cdot ({\rho }_{\alpha }{v}_{\alpha })+{\rho }_{\alpha }{q}_{\alpha }$$

*S* is the saturation of the phase, *ϕ* is the porosity of the rock, *ρ* is the density of the phase, *q* is the source-sink rate of the phase, *v* is the Darcy velocity for phase, and *t* is time. We also assume capillarity and gravity forces are negligible. The Darcy phase velocity is,

2 $${v}_{\alpha }=-{\lambda }_{\alpha }K\nabla {P}_{\alpha }$$

where *P* is the phase pressure, *K* is the absolute rock permeability, and *λ* is the phase mobility,

3 $${\lambda }_{\alpha }=\frac{{k}_{r\alpha }({S}_{\alpha })}{{\mu }_{\alpha }}$$

where *k*_*r*_ is the relative permeability of phase and *μ* is fluid viscosity of phase. The fluid viscosity is constant and usually different for each phase. The relative permeability is saturation dependent, and in this paper we use Corey-type^41^ functions,

4 $${k}_{ro}={(1-{S}_{wo})}^{{N}_{o}},\quad {k}_{rw}={k}_{rw}^{o}{S}_{wo}^{{N}_{w}}$$

5 $${S}_{wo}=\frac{{S}_{w}-{S}_{{w}_{i}}}{1-{S}_{{w}_{i}}-{S}_{or}}$$

where *N*_*o*_ and *N*_*w*_ are history matching parameters, ${k}_{rw}^{o}$ is the end point of the water relative permeability, *S*_*wo*_ is the normalized water saturation, ${S}_{{w}_{i}}$ is initial water saturation and *S*_*or*_ is residual oil saturation.

In our simulation, we assume a closed system with know boundary conditions, and flowing source/sink terms represented by wells. For the cases when incompressible fluids are assumed, we can write a pressure equation that is independent of the water saturation of the system^25^.

The pressure equation is,

6 $$\phi {C}_{r}\frac{\partial P}{\partial t}+\nabla \cdot v={q}_{t}$$

where *C*_*r*_ is rock compressibility, *q*_*t*_ is the total source-sink rate of both phases and *v* is the total velocity,

7 $$v=-{\lambda }_{t}K\nabla P$$

where *λ*_*t*_ is the total mobility given by,

8 $${\lambda }_{t}=\frac{{k}_{ro}(1-{S}_{w})}{{\mu }_{o}}+\frac{{k}_{rw}({S}_{w})}{{\mu }_{w}}$$

We then calculate the velocity of the phase using Darcy’s law. Equation (2) and the advection transport of fluid, Eq. (1). The pressure and advection equations are then coupled non-linearly and the total mobility (saturation dependent over time and space) is upstreamed, Eq. (8).

## Numerical method

In the NCFVE method, a multi-phase flow problem is solved in two steps. First, the primary variable, pressure, is calculated using the finite element method; in this paper we use the well-established Galerkin method^12,37,39^ Then, the advection of fluid between the node control volumes is calculated using the finite volume method. In this paper, we do not detail the discretization procedures of the governing equations and the construction of the secondary control volume mesh, as they are thoroughly discussed by Abushaikha *et al*.^37^. We rather present the final form of the pressure equation:

9 $$\phi {C}_{r}\frac{\partial P}{\partial T}-\nabla .{\bf{K}}{\lambda }_{t}\nabla P-\nabla .{\bf{K}}{\lambda }_{w}\nabla {P}_{c}-{\bf{K}}({\lambda }_{w}{\rho }_{w}+{\lambda }_{o}{\rho }_{o})\nabla {\bf{g}}={q}_{t}$$

There are five finite element matrices that need to be defined for each element based on the recovery mechanisms in the pressure equation:

1. Conductance matrix [M].
2. Capillarity matrix [CP].
3. Gravity matrix [G].
4. Capacitance matrix [C].
5. Source/sink terms (force vector) [F].

We use implicit pressure and explicit saturation (IMPES) so Eq. (9) becomes:

10 $$({[C]}^{t}+\bigtriangleup t{[M]}^{t}){[P]}^{t+1}={[C]}^{t}{[P]}^{t}+\bigtriangleup t({[F]}^{t+1}+{[CP]}^{t}-{[G]}^{t})$$

where *t* is the time-step. The former equation is an *Ax* = *b* equation and we solve for the unknown, the next time-step of pressure [*P*]^*t*+1^, using the Generalized Minimum Residual (GMRES) linear solver with final residual of 1.0 × 10^−9^^42^.

After the pressure is calculated, we integrate the transport equation over the node control volume (*n*) and apply the divergence theorem and the forward Euler discretization in time to get:

11 $${S}_{w,(n)}^{t+1}=\frac{{S}_{w,(n)}^{t}C{V}^{t}+\bigtriangleup t\left[-\mathop{\sum }\limits_{j}^{SI}flu{x}_{(n),j}+{q}_{(n),w}\right]}{C{V}^{t+1}}$$

where *CV* is the pore volume *V*_(*n*)_, area *A*_(*n*)_, or line *L*_(*n*)_ depending on the type of node control volume mesh (it is pressure dependent: updated each time step using equation $\phi ={\phi }_{i}{e}^{{C}_{r}(P-{P}_{i})}$, SI is the number of faces in node control volume (*n*), and the *flux*_(*n*),*j*_ is the flux of face *j* in node control volume (*n*) and calculated by,

12 $$flu{x}_{(n),j}={({\lambda }_{w}^{{t}^{(e)}}{K}^{(e)}\nabla {\phi }^{(e)})}_{(n),j}.{{\bf{N}}}_{(n),j}$$

where **N** is the are normal vector.

We are only interested in the new saturation values applied in Eq. (8) to update the pressure equation, Eq. (9). We discuss this in the next section.

## Upstream mobility calculation (UMC) for NCVFE method

**Figure 2:**
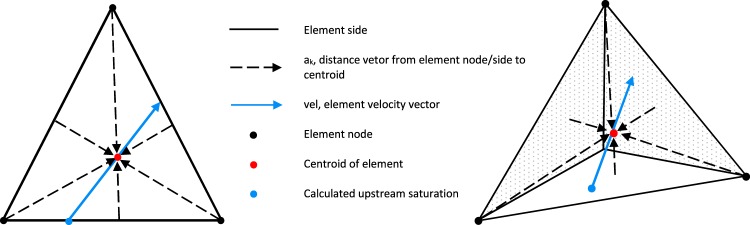
Equation (9) parameters for: triangle (left), tetrahedron (right). The blue dot is the calculated upstream saturation for the element.

Using the upstream node control volume, similar to the finite volume method, for the mobility calculation in Eq. (8) for the pressure Eq. (6) produce unphysical fluid saturation profiles, as discussed by Abushaikha^43^. In this paper, we introduce an equation to allocate the upstream direction of the fluid flow over each element. It uses the element velocity vector and a weighting procedure to determine the saturation at the point where the tail of velocity vector intersects the element, see Fig. 2. The equation is given below,

13 $${S}_{upstream}=\frac{\mathop{\sum }\limits_{k=1}^{F}{A}_{k}{S}_{k}}{\mathop{\sum }\limits_{k=1}^{F}{A}_{k}}$$

where *S*_*upstream*_ is the calculated upstream water saturation for element, *F* is the number of interfaces and nodes for element (equals 6 for a triangle and 8 for a tetrahedron), *S*_*k*_ is the water saturation at location K for element, and *A*_*k*_ is given by,

14 $${A}_{k}=max[0,{a}_{k}\cdot vel]$$

where *A*_*k*_ is the component of the distance vector projected onto the velocity vector, if this dot product is positive (no obtuse angle) its saturation is accounted for, otherwise it is not, *vel* is the element velocity and *a*_*k*_ the distance vector from location k (node or centroid of interface) to element centroid, see Fig. 2. This equation is used to compute Eq. (8) to be used in Eq. (6).

The algorithm below shows the implementation steps for NCVFE numerically.

**Algorithm 1:**
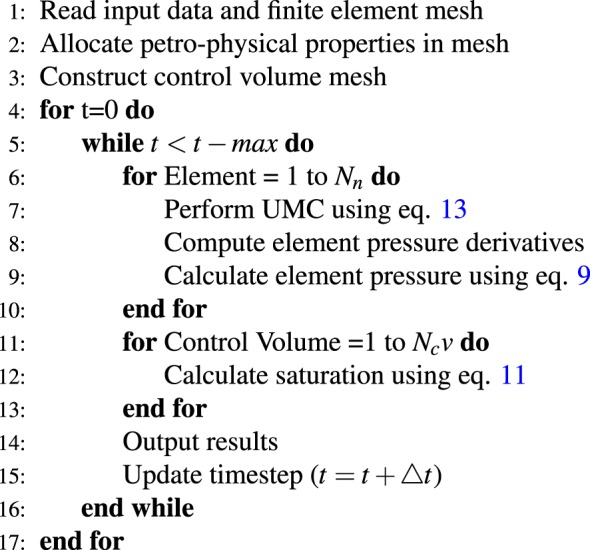
The algorithm for NCVFE.

## Numerical tests

In this section we test the UMC in the NCVFE method for various cases. In all the tests, water and oil viscosities are 0.4 and 2.5 mPa.s respectively and the rock compressibility equals 4.0 × 10^−10^*Pa*^−1^. We assume the medium is fully saturated with oil and the porosity and permeability are 0.2 and 100 mD respectively, unless otherwise stated.

### Validation: Buckley-Leverett problem

**Figure 3:**
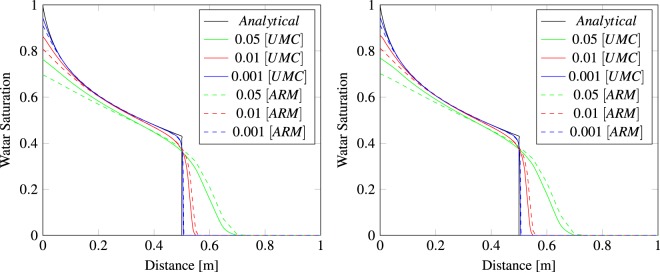
Comparison of the analytical solution of the Buckley-Leverett problem at distance 0.5 m from the left-hand boundary to the numerical solution of the NCVFE method using three meshes for triangles (left) and tetrahedron (right) elements. Water is injected at the left and displaces oil in a homogeneous porous medium.

To validate the new upstream mobility calculation for the NCVFE method, we use the one dimensional analytical solution of the Buckley-Leverett problem, where gravity and capillarity are negligible^4,26^. The domain is represented by a plane with dimensions of 1 m  ×  Element Length to test the triangle elements and by a rectangular tube with dimensions of 1 m  ×  Element Length  ×  Element Length for the tetrahedron elements. Figure 3 compares the analytical solution to the numerical solutions of the traditional approach (arithmetic weighting) versus the UMC method for the two types of domains using different element lengths. We can see the UMC method produces a more confined saturation profiles with a shaper front than the traditional approach for both types of elements.

**Figure 4:**
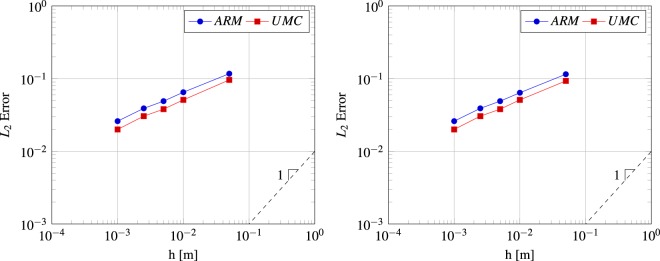
Convergence of the *L*_2_ error of water saturation as a function of the mesh element length for the numerical solutions of the Buckley-Leverett problem using the 3-D tetrahedral (left) and @-d traingular (right) elements.

To measure the difference between the two methods, we calculate the error L2 norm for the saturation profiles for various element lengths (also to measure the rate of convergence). Figure 4 shows this error for triangle and tetrahedron elements, respectively (both elements produce almost identical saturation profiles from this problem). The UMC method produces less error for the same element length than the traditional approach for this validation test. Also, both methods produce a sub-linear convergence rate of approximately 0.4. These relatively modest convergence rates are a result of the shock between the two fluids that dominates the overall error. Schmid *et al*.^39^ and Hoteit and Firoozabadi^44^ applied the Buckley-Leverett problem for various numerical methods. They observed low convergence rates for the Buckley-Leverett problem regardless of the numerical method used, as we saw here, because of this sharp separation between the two fluids.

In this test, we validated and tested the convergence of the traditional approach and the new method for triangle and tetrahedron elements. We saw the traditional approach not producing very accurate saturation profiles because the fluid mobility needed for the pressure calculation is computed using the arithmetic weighting of the encompassed control volumes saturations. On the other hand, the UMC method employs the velocity vector to allocate the upstream saturation therefor producing more accurate saturation profiles. Next, we test the method for multi-dimensional domains.

### Homogeneous test: five spot case

**Figure 5:**
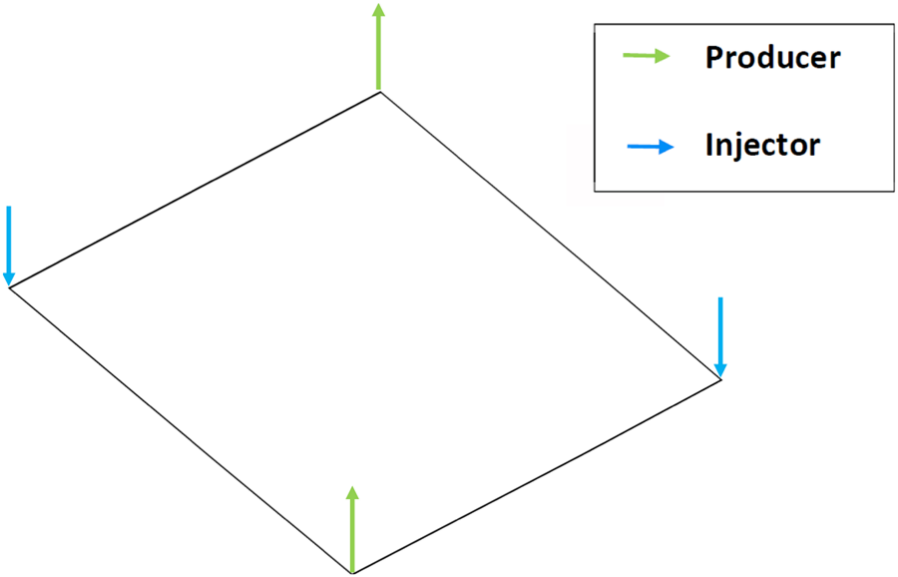
The domain of the homogeneous case with producers and injectors at the corners.

The five-spot is a simple case of a water flooding scenario in a homogeneous domain. Water is injected at constant rate in the top left and lower right corners, while oil (including water after breakthrough) is extracted at the same constant rate in the lower left and upper right corners. The domain is represented by a plane with dimensions of 1 m  ×  1 m to test the triangle elements and by a cube with dimensions of 1 m  ×  1 m  ×  Element Length for the tetrahedron elements (see Fig. 5). In this test, we use two meshes (coarse and fine) for each domain and test the UMC method and the traditional approach (arithmetic weighting). The mesh properties are listed in Table 1.

**Table 1:** The mesh properties of Homogeneous test.

Element type	Mesh	Elements	Nodes
Triangle	Coarse	242	142
	Fine	26,528	13,465
Tetrahedron	Coarse	881	325
	Fine	82,538	27,556

**Figure 6:**
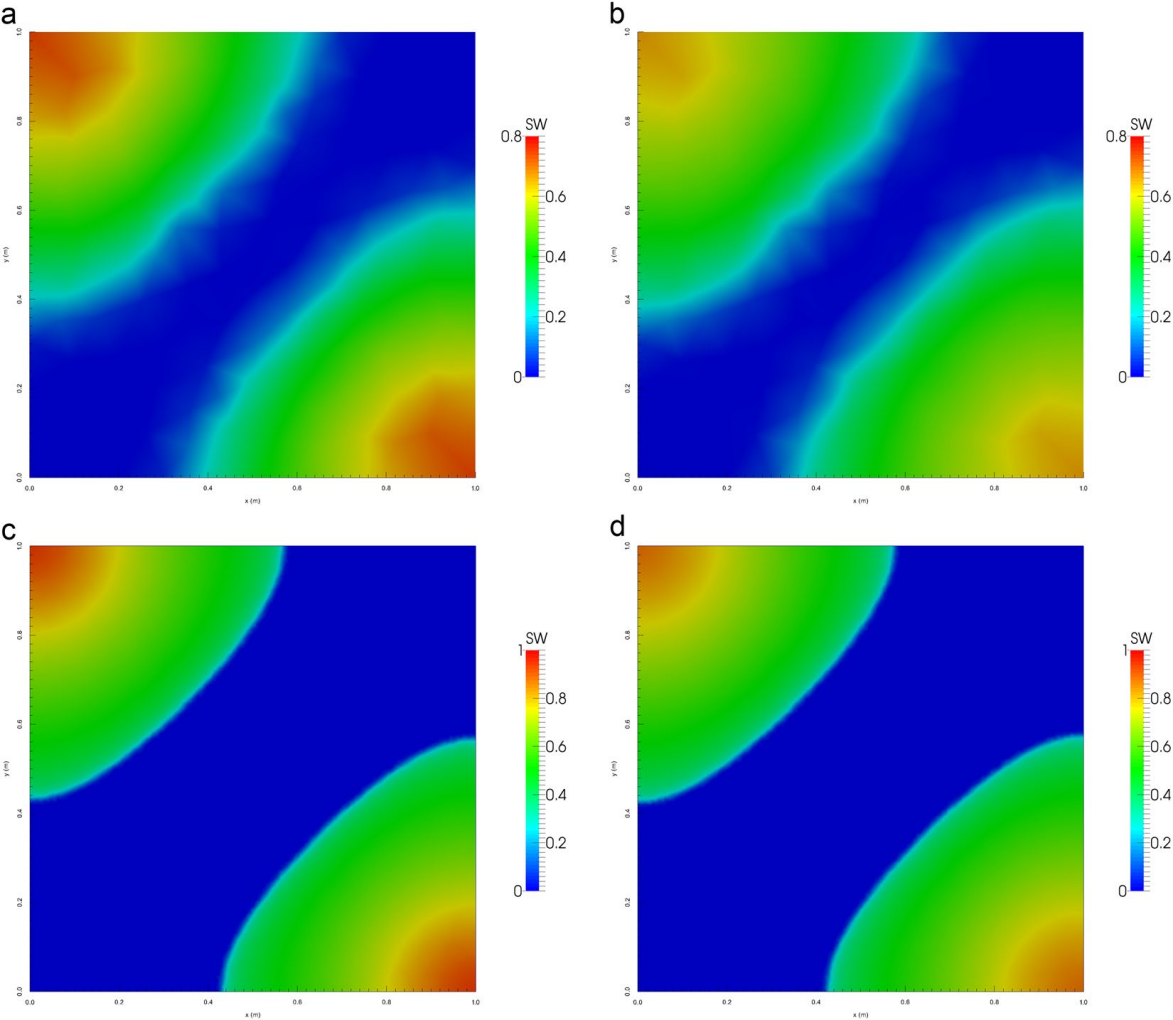
Water saturation field modelled using ARM (left) and UMC (right) approaches for mobility calculations using a coarse and a fine mesh for triangular elements (2-D).

 Figure 6 show the water saturation profiles after pore volumes of water have been injected in the domains composed of triangles and tetrahedrons. We see the water is more confined and the front is less smeared when applying the UMC method for both domains and mesh resolutions versus the traditional approach. To analyse this behaviour, we calculate the water cut at the oil producers as a function of pore volumes injected to measure the breakthrough time for both methods. The water cut is given by

15 $$WC=\frac{{q}_{w}}{{q}_{t}}$$

where *q*_*t*_ = *q*_*w*_ + *q*_*o*_, *q*_*w*_ and *q*_*o*_ equal the water and oil production flow rates.

**Figure 7:**
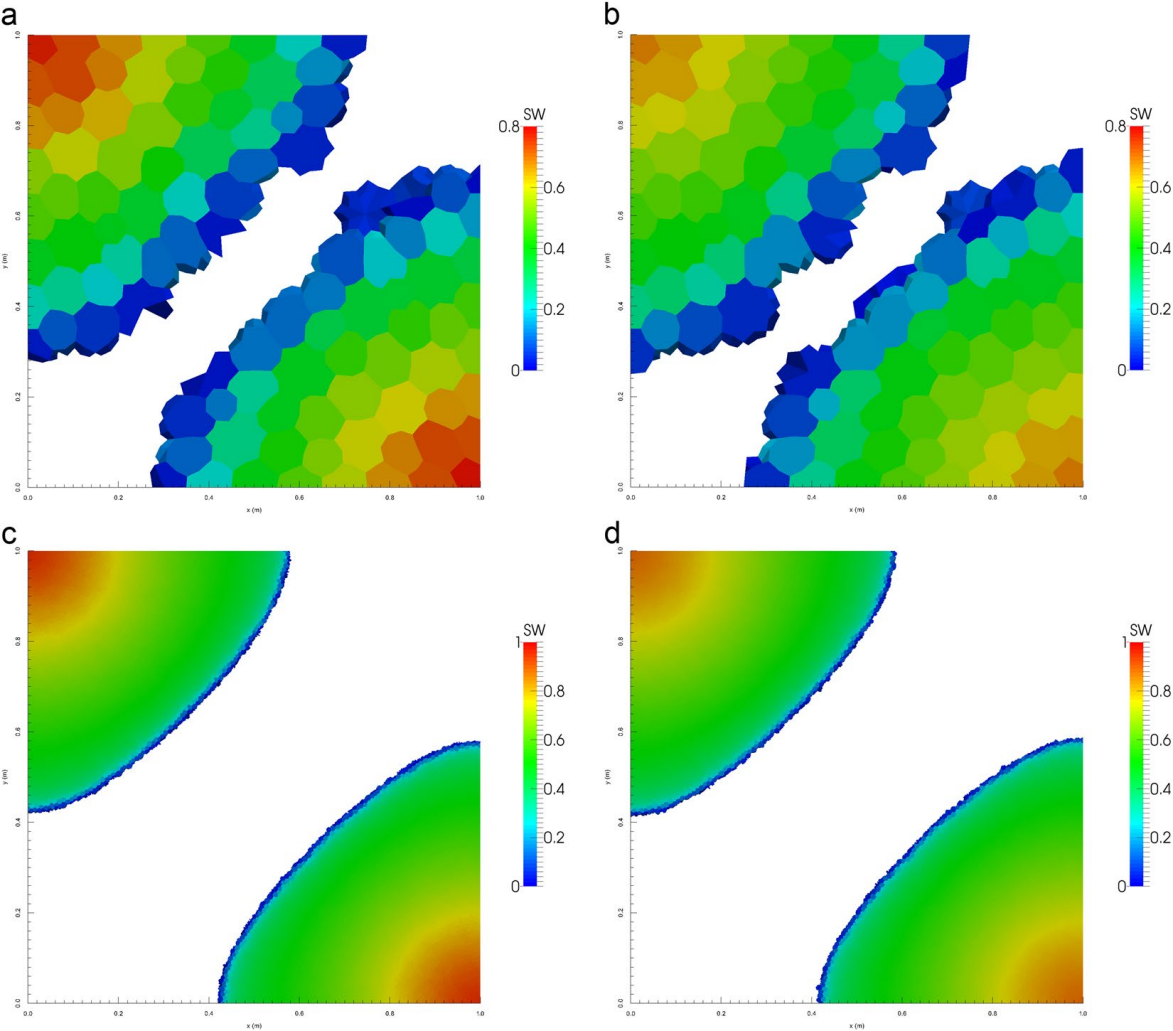
Water saturation field modelled using UMC (left) and ARM (right) approaches for mobility calculations using a coarse and a fine mesh for tetrahedral elements (3-D).

**Figure 8:**
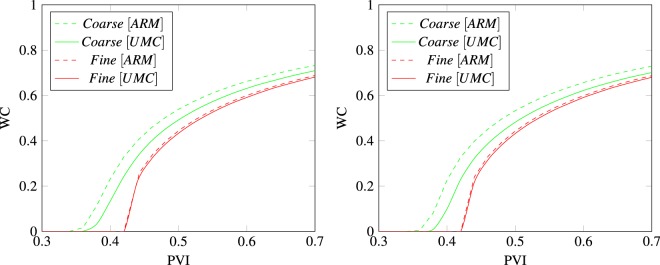
Comparison of the water cut profiles per injected pore volume for triangular (left) and tetrahedral (right) elements for the mesh sizes seen in Table 1.

 Figures 6 and 7 show the water breakthrough times at the oil producers for the domains composed of triangles and tetrahedrons. Moreover, we observe in Fig. 8 that the breakthrough is delayed when applying the UMC method and water reaches the oil producers sooner when using the traditional approach. We can also see the effect of grid orientation where fine meshes produce a high resolution of the water front and a delay of the breakthrough time versus the coarse meshes for both domains. This behaviour is in agreement with the previous validation test (Buckley -Leverett) as the UMC method produces a more confined water saturation profiles with a sharper front delaying the breakthrough time.

Next, we test the UMC method in more heterogeneous domains represented by highly conductive zones (fractures).

### Heterogeneous domain

**Figure 9:**
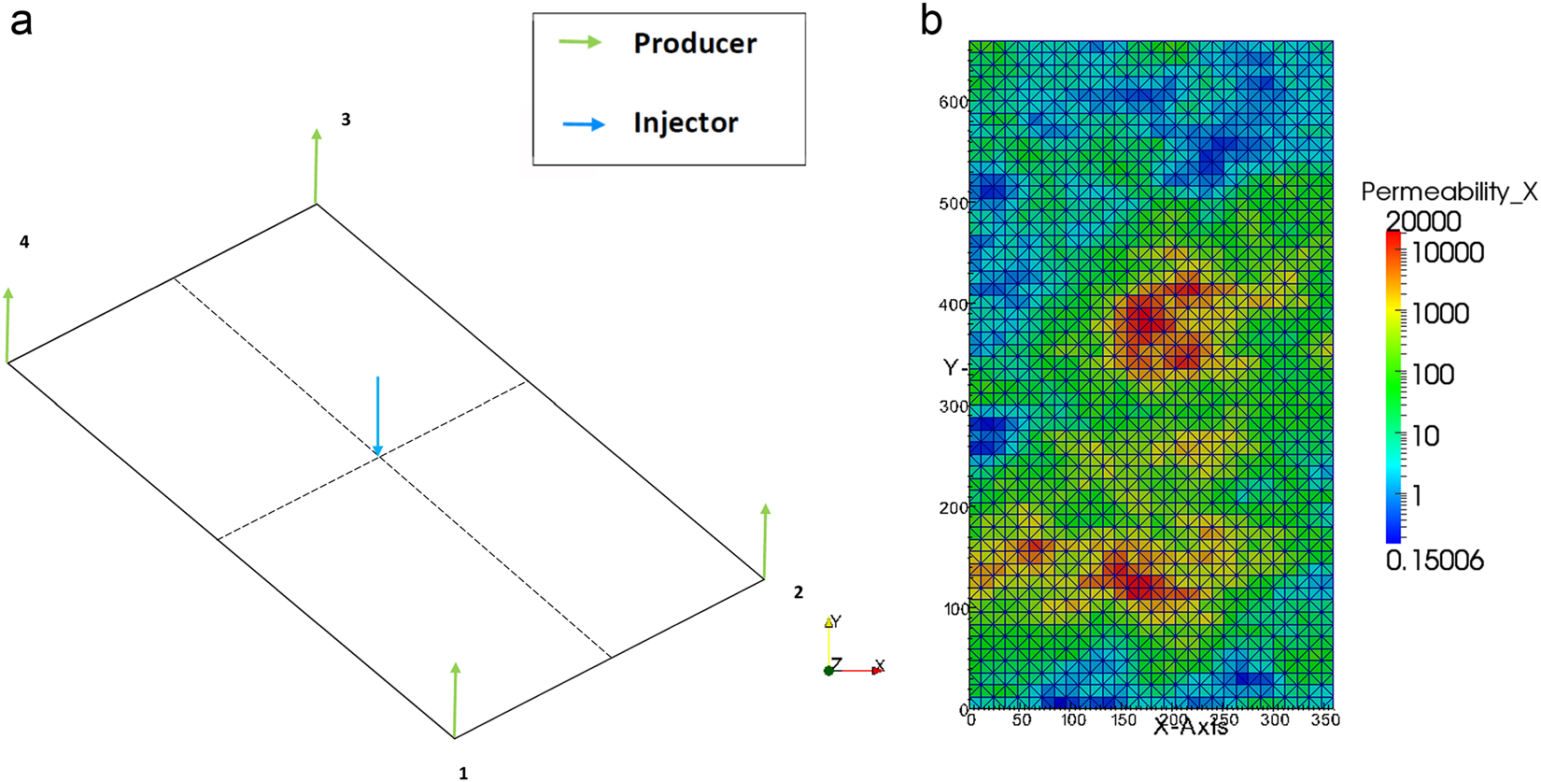
The figure in (**a**) shows the domain with injectors and producers while (**b**) shows the heterogeneous permeability of the domain.

In this last test, a heterogeneous petro-physical model of Coats Engineering upscaled model of SPE10 layer 10^45^ The area is meshed in 2-D using triangular elements with a central water injector that pushes the oil into the four producers located at the corners as shown in Fig. 9. The properties of the mesh used in this test are detailed in Table 2.

**Table 2:** The mesh properties of the heterogeneous test.

Element type	Mesh	Elements	Nodes	Grid/Element size (*m*^2^)
Triangle	Fine	3300	1736	72

**Figure 10:**
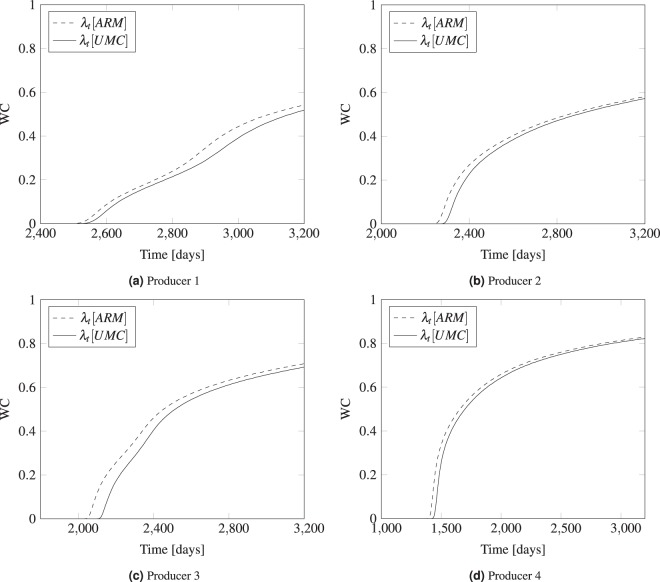
Plots of water cut vs. time (days) comparing the ARM and UMC mobility approaches in mobility estimation for the four oil producers.

The water cut for the producers is plotted in Fig. 10 against the production time using Eq. (15). The results are compared for the arithmetic and UMC approach of estimating the mobility. It is noticed that the water cut profiles, when arithmetic mobility is used, lag behind the UMC curve in the four producers. This observation is consistent with the results of the homogeneous test where the breakthrough of water is delayed with UMC. The breakthrough point of water is different at each injector despite the fact the injector is placed in the center at equal distance from the producers. This could be explained by the highly heterogeneous layer that produces varying pressure responses at different locations due to permeability variations. Hence, the direction of the flow will be skewed towards the region with high permeability, and thus different breakthrough times are noticed.

This velocity up-winding scheme is very crucial when up-scaling techniques are implemented. A solid understanding of the flow functions governing fluid flow in fractured reservoirs provides the necessary foundation for up-scaling laboratory results to the field scale using numerical simulators. Scaling groups are mainly used to increase computational efficiency of simulators by several orders of magnitudes. However, truncation error in up-scaling is inevitable, and the up-winding of the velocity using UMC can contribute to improving the saturation calculation.

## Summary and Conclusions

In this work, we have presented a novel approach for up-streaming mobility calculation in NCVFE simulators for triangular (2-D) and tetrahedral (3-D) domains. Based on our results, we were able to show that our method produces more accurate fluid saturation profiles compared to the traditional (arithmetic) approach. The utilization of the velocity vector to determine the appropriate saturation for upstreaming is more accurate than the arithmetic weighting of the saturations in the control volumes. Upon injection of water in a simulation case, the water front was less smeared when UMC is used for different mesh resolutions and the breakthrough of water is delayed. Moreover, the UMC approach can show good accuracy when the method is used for up-scaled models, up-scaling is an essential approach when the aim is to reduce simulation time and cost, and UMC allows for better prediction of the movement of the water front in such scenarios.

This novel approach can be easily implemented in current reservoirs simulators and has a wide range of applicability to various fields of hydrocarbon recovery, ground water movement and contaminant transport.

## Appendix A

We explicitly define the five finite element matrices of the pressure equation (10).

[*M*^(*e*)^] is the conductance matrix, and for tetrahedron element equals,

A-1 $${[{M}^{(e)}]}_{n\times n}={(K{\lambda }_{t})}^{(e)}{V}^{(e)}\left[\begin{array}{ccc}\frac{\partial {N}_{1}^{(e)}}{\partial x} & \frac{\partial {N}_{1}^{(e)}}{\partial y} & \frac{\partial {N}_{1}^{(e)}}{\partial z}\\ \vdots  & \vdots  & \vdots \\ \frac{\partial {N}_{n}^{(e)}}{\partial x} & \frac{\partial {N}_{n}^{(e)}}{\partial y} & \frac{\partial {N}_{n}^{(e)}}{\partial z}\end{array}\right]\ \left[\begin{array}{ccc}\frac{\partial {N}_{1}^{(e)}}{\partial x} & \ldots \  & \frac{\partial {N}_{n}^{(e)}}{\partial x}\\ \frac{\partial {N}_{1}^{(e)}}{\partial y} & \ldots \  & \frac{\partial {N}_{n}^{(e)}}{\partial y}\\ \frac{\partial {N}_{1}^{(e)}}{\partial z} & \ldots \  & \frac{\partial {N}_{n}^{(e)}}{\partial z}\end{array}\right]$$

[*CP*^(*e*)^] is the capillarity matrix,

A-2 $${[C{P}^{(e)}]}_{n\times n}={(K{\lambda }_{t})}^{(e)}{V}^{(e)}\left[\begin{array}{ccc}\frac{\partial {N}_{1}^{(e)}}{\partial x} & \frac{\partial {N}_{1}^{(e)}}{\partial y} & \frac{\partial {N}_{1}^{(e)}}{\partial z}\\ \vdots  & \vdots  & \vdots \\ \frac{\partial {N}_{n}^{(e)}}{\partial x} & \frac{\partial {N}_{n}^{(e)}}{\partial y} & \frac{\partial {N}_{n}^{(e)}}{\partial z}\end{array}\right]\ \left[\begin{array}{ccc}\frac{\partial {N}_{1}^{(e)}}{\partial x} & \ldots \  & \frac{\partial {N}_{n}^{(e)}}{\partial x}\\ \frac{\partial {N}_{1}^{(e)}}{\partial y} & \ldots \  & \frac{\partial {N}_{n}^{(e)}}{\partial y}\\ \frac{\partial {N}_{1}^{(e)}}{\partial z} & \ldots \  & \frac{\partial {N}_{n}^{(e)}}{\partial z}\end{array}\right]\ \left[\begin{array}{c}{P}_{C,i}\\ {P}_{C,j}\\ {P}_{C,k}\\ {P}_{C,l}\end{array}\right]$$

[*G*^(*e*)^] is the gravity matrix,

A-3 $${[{G}^{(e)}]}_{n\times 1}=({\rho }_{w}{\lambda }_{w}+{\rho }_{o}{\lambda }_{o})g{V}^{(e)}\left[\begin{array}{c}\frac{\partial {N}_{1}^{(e)}}{\partial z}\\ \vdots \\ \frac{\partial {N}_{n}^{(e)}}{\partial z}\end{array}\right]$$

[*C*^(*e*)^] is the capacitance matrix,

A-4 $${[{C}^{(e)}]}_{n\times n}=\phi {C}_{r}\frac{1}{n}{V}^{(e)}{\left[\begin{array}{ccc}1 &  & 0\\  & \ddots  & \\ 0 &  & 1\end{array}\right]}_{n\times n}$$

[*F*^(*e*)^] is the force vector,

A-5 $${[{F}^{(e)}]}_{n\times 1}={V}^{(e)}{q}_{t}{\left[\begin{array}{c}{N}_{1}^{(e)}({x}_{0},{y}_{0},{z}_{0})\\ \vdots \\ {N}_{n}^{(e)}({x}_{0},{y}_{0},{z}_{0})\end{array}\right]}_{n\times 1}$$

**Figure 11:**
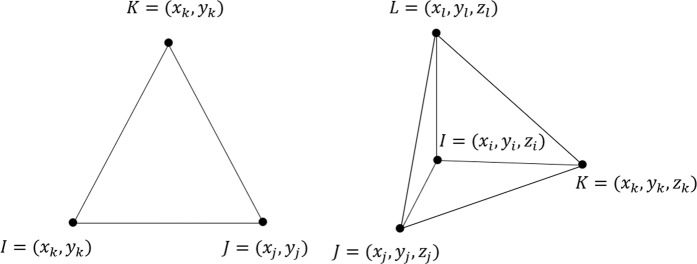
Linear finite elements: triangle (left), and tetrahedron (right).

The interpolation function *N* and their derivatives are defined for a triangle and tetrahedron as follows (see Fig. 11).

**Triangles**

A-6 $${N}_{i}^{(e)}=\frac{1}{2{A}^{(e)}}({a}_{i}+{b}_{i}x+{c}_{i}y),\ \ \frac{\partial {N}_{i}^{(e)}}{\partial x}=\frac{{b}_{i}}{2{A}^{(e)}},\ \ \frac{\partial {N}_{i}^{(e)}}{\partial y}=\frac{{c}_{i}}{2{A}^{(e)}}$$

A-7 $${N}_{j}^{(e)}=\frac{1}{2{A}^{(e)}}({a}_{j}+{b}_{j}x+{c}_{j}y),\ \ \frac{\partial {N}_{j}^{(e)}}{\partial x}=\frac{{b}_{j}}{2{A}^{(e)}},\ \ \frac{\partial {N}_{j}^{(e)}}{\partial y}=\frac{{c}_{j}}{2{A}^{(e)}}$$

A-8 $${N}_{k}^{(e)}=\frac{1}{2{A}^{(e)}}({a}_{k}+{b}_{k}x+{c}_{k}y),\ \ \frac{\partial {N}_{k}^{(e)}}{\partial x}=\frac{{b}_{k}}{2{A}^{(e)}},\ \ \frac{\partial {N}_{k}^{(e)}}{\partial y}=\frac{{c}_{k}}{2{A}^{(e)}}$$

where

$$\begin{array}{lll}{a}_{i}={x}_{j}^{(e)}{y}_{k}^{(e)}-{x}_{k}^{(e)}{y}_{j}^{(e)} & {a}_{j}={x}_{k}^{(e)}{y}_{i}^{(e)}-{x}_{i}^{(e)}{y}_{k}^{(e)} & {a}_{k}={x}_{i}^{(e)}{y}_{j}^{(e)}-{x}_{j}^{(e)}{y}_{i}^{(e)}\\ {b}_{i}={y}_{k}^{(e)}-{y}_{k}^{(e)} & {b}_{j}={y}_{k}^{(e)}-{y}_{i}^{(e)} & {b}_{k}={y}_{i}^{(e)}-{y}_{j}^{(e)}\\ {c}_{i}={x}_{k}^{(e)}-{x}_{j}^{(e)} & {c}_{j}={x}_{i}^{(e)}-{x}_{k}^{(e)} & {c}_{k}={x}_{j}^{(e)}-{x}_{i}^{(e)}\end{array}$$

**Tetrahedrons**

A-9 $${N}_{i}^{(e)}=\frac{Volum{e}_{{\bf{XJKL}}}}{Volum{e}_{{\bf{IJKL}}}}=\frac{({\bf{J}}-{\bf{X}})\cdot (({\bf{K}}-{\bf{J}})\times ({\bf{L}}-{\bf{J}}))}{({\bf{I}}-{\bf{X}})\cdot (({\bf{K}}-{\bf{J}})\times ({\bf{L}}-{\bf{J}}))}$$

A-10 $${N}_{j}^{(e)}=\frac{({\bf{K}}-{\bf{X}})\cdot (({\bf{L}}-{\bf{K}})\times ({\bf{I}}-{\bf{K}}))}{({\bf{K}}-{\bf{J}})\cdot (({\bf{L}}-{\bf{K}})\times ({\bf{I}}-{\bf{K}}))}$$

A-11 $${N}_{k}^{(e)}=\frac{({\bf{L}}-{\bf{X}})\cdot (({\bf{I}}-{\bf{L}})\times ({\bf{J}}-{\bf{L}}))}{({\bf{L}}-{\bf{K}})\cdot (({\bf{I}}-{\bf{L}})\times ({\bf{J}}-{\bf{L}}))}$$

A-12 $${N}_{l}^{(e)}=\frac{({\bf{I}}-{\bf{X}})\cdot (({\bf{J}}-{\bf{I}})\times ({\bf{K}}-{\bf{I}}))}{({\bf{I}}-{\bf{L}})\cdot (({\bf{J}}-{\bf{I}})\times ({\bf{K}}-{\bf{I}}))}$$

A-13 $$\frac{\partial {N}_{i}^{(e)}}{\partial x}=\frac{-{(({\bf{K}}-{\bf{J}})\times ({\bf{L}}-{\bf{J}}))}_{x}}{({\bf{I}}-{\bf{X}})\cdot (({\bf{K}}-{\bf{J}})\times ({\bf{L}}-{\bf{J}}))}$$

A-14 $$\frac{\partial {N}_{j}^{(e)}}{\partial x}=\frac{-{(({\bf{L}}-{\bf{K}})\times ({\bf{I}}-{\bf{K}}))}_{x}}{({\bf{K}}-{\bf{J}})\cdot (({\bf{L}}-{\bf{K}})\times ({\bf{I}}-{\bf{K}}))}$$

A-15 $$\frac{\partial {N}_{k}^{(e)}}{\partial x}=\frac{-{(({\bf{I}}-{\bf{L}})\times ({\bf{J}}-{\bf{L}}))}_{x}}{({\bf{L}}-{\bf{K}})\cdot (({\bf{I}}-{\bf{L}})\times ({\bf{J}}-{\bf{L}}))}$$

A-16 $$\frac{\partial {N}_{l}^{(e)}}{\partial x}=\frac{-{(({\bf{J}}-{\bf{I}})\times ({\bf{K}}-{\bf{I}}))}_{x}}{({\bf{I}}-{\bf{L}})\cdot (({\bf{J}}-{\bf{I}})\times ({\bf{K}}-{\bf{I}}))}$$

where

$${\bf{I}}=({x}_{i},{y}_{i},{z}_{i})$$

$${\bf{J}}=({x}_{j},{y}_{j},{z}_{j})$$

$${\bf{K}}=({x}_{k},{y}_{k},{z}_{k})$$

$${\bf{L}}=({x}_{l},{y}_{l},{z}_{l})$$
